# London's Problem Solved

**Published:** 1887-12-03

**Authors:** 


### The Hospital

A Weekly Institutional Journal of

**Science, Medicine, and Philanthropy.**

*For Hospitals, Asylums, Medical Practitioners, Students, Nurses, Families, Parishes, Congregations, and the Charitable Public.*

Vol. III. No. 62.] [December 3, 1887.

### London's Problem Solved.

One striking result of the development of "specialism" in recent years has been to show how well things can be done when men, goaded by the spur of competition, give their undivided energies to one particular thing. So that now, he who has means to pay for the very best product of skill or the very best quality of work, always insists upon having it, and, practically, may always get it. From the individual employed and paid by the individual to the servants of the community employed and paid by the community is but a step, and an easy step; and similarly from the paid servants of the Commonwealth to its elected representatives and rulers it is but a step. Those paid servants and those elected representatives are "specialists," paid and selected to do certain work, and to do it as well as it can be done. If they do not do it as well as it can be done, the modern impatience with unskilled labour must get rid of both incompetent paid servants and incapable elected representatives, and try others—and continue trying until capable ones are found. This, of course, does not refer to the present or any particular political party which may be in office at the moment, but applies to each political party, and to all public servants of whatever rank or degree.

We are now concerned with one large and important branch of the public service which, in many respects, has of late years been as incompetently administered as it could well have been. Many of its methods have been bad, and the results bad; whilst the cost has been shameful. We refer to the public care and management of the unemployed and the pauper population, more especially in the metropolis. The justification of these assertions will be found in the special *supplement* which we publish this week; and, therefore, we need not stop here to argue the question. What it is desired to do, however, is to ask, with as much plainness and point as may be possible, whether or not we as a community are at length ashamed of ourselves and our dreadful "residuum," and whether or not the time has arrived, and is now here, when we should set about mending or ending the whole business of our poor-law administration root and branch.

What ought to be done with the unemployed and paupers? We know what has been done. They have been treated as a hateful burden, an unmitigated nuisance, a wretched army of parasites sucking the juices out of the social organism—parasites of which no possible use could be made, and which we only did not kill and destroy because, being human beings of more or less conscience and education, we could not bring ourselves to do so. But that treatment has failed, failed entirely, as it deserved to fail. It was not reasonable, and it was not human; and, therefore, it carried within itself the seeds and elements of failure. This broad general principle must always remain true and final, that in whatever dealings a man has with his brother man, he must never eliminate the element of humanity. Men must always, and in all circumstances, be treated as men and not as brute beasts, or as things without life. If they are not, the Nemesis of outraged humanity is at once upon the track of the stupid or wicked fools who have ignored this fundamental principle, and sooner or later the day of reckoning dawns.

Our day of reckoning has at length dawned. We are face to face with a state of things which can no longer be tolerated, and which is as dangerous as it is intolerable. The problem for solution is like many other problems, difficult to the incapable and impossible to the indifferent. But given a will to solve it and average capacity, the thing can be done and done effectually. The idea that the poor and the unemployed are mere parasites must be got rid of at once and completely. All helpless things are not parasites. Children are helpless for many years, but they are not parasites; soldiers and sailors are often unemployed, but they are not parasites. The only human beings who may properly be called parasites are the irreclaimable criminals. The merely poor and the unemployed belong to a totally different class. They must for the future be regarded as instruments, human instruments, which can be put to a certain profitable use. Once let that idea lay hold of the governing minds in the State, and all kinds of wise and beneficial results will flow from it. But the idea must take firm root; it must be held as a fundamental conviction, that human beings who are possessed of some physical powers and retain a degree of moral sense are capable of rendering intelligent and profitable service to the Commonwealth.

The first step in the solution of the problem is to resolve that all the poor and unemployed who are capable of work, in however limited a degree, shall be furnished with the opportunity of working—work shall be found for them. But it must be *bonâ fide* useful and productive work, not penal labour. The idea of labour which is merely penal, and in itself unproductive, is absurd as applied to many of the poor and unemployed. It is punishing them, not for crime but for misfortune. It is not merely stupid, it is immoral. When misfortune has hunted a man down and is holding him fast by the throat, then comes his brother man, and instead of helping, gives him a contemptuous kick and consigns him to the "Inferno" of degraded and worthless things. The State, that is the Government which is at the moment in power, should and must find work for the unemployed. We need not now discuss the merely academic question of the abstract functions of Governments in these matters. That would be futile, childish, silly. Here are tens of thousands of unemployed and starving poor at our doors. Have we any profitable work which they can do? And if we have not, can we make any? We do not need to make any. Here is the work ready to hand as well as the workmen, and it only needs the word of authority, the officiating priest, to make the marriage between the workers and the work an accomplished fact.

As everybody knows and has known for years a Royal Commission has decided that the London sewage now conducted to Barking Creek is a source of great and growing danger to the whole population of this vast and increasing metropolis. As dealt with now the sewage as manure is lost and wasted, whilst its presence in the river is destructive alike to purity and beauty as well as being poisonous to its fish and to the people along its banks. That the river ought to be purified is an axiom among all men who have the merest element of common sense; that it is not purified fills one with contempt for those who have been responsible for the conduct of metropolitan affairs. What is the use of rulers who cannot rule and administrators who cannot administer? Is there no sense of proportion among our legislators? They strain at gnats and swallow, not camels merely, but church steeples, and whole cathedrals even. What work have they had in hand of late years which can compare in practical importance with the purification of the Thames? Here is a ten years' period of employment for all the destitute of London, which would confer an incalculable advantage upon every other class of its inhabitants, besides enriching all the soil along the line of the sewage from its present outfall to the open sea. No more words need be wasted on the topic. There it is; it remains for the Government to say the word or leave the matter alone. For half-a-million of money the works could be initiated; and every unemployed man and boy in London could be furnished with productive and profitable labour forthwith.

Such is the workable scheme which lies ready to hand for the unemployed of London. For those who have no work in provincial towns and outlying districts the *home colonisation* project propounded and so ably elaborated by Mr. Herbert Mills and approved by many other thoughtful and practical people is ripe for experiment. That it also is practicable and in every way immediately desirable no one who is familiar with agricultural pursuits, and who has taken note of the vast acreage of land which is going out of cultivation in this country, for a moment doubts. Similar schemes are in operation in Germany, Belgium, and elsewhere, and have been successful. But even if they were not, what kind of miserable cowardice is it which, at a crisis like the present, stands shivering with dread, and refuses to try a thing because it is new, and may lead to the loss of a few thousand pounds? Our wealthier people and rulers are surely all in their dotage when they present such spectacles of feeble courage and irresolute will!

Here, then, is the problem solved. Give profitable work to the unemployed, and supplement it by an efficient emigration scheme. If the Government will take the matter in hand the classes need no longer fear the masses, and the masses will no longer hate the classes. If Members of Parliament and the wealthier inhabitants of their boroughs will give thought and attention to the scheme, it may be carried out thoroughly and in detail. The subject which now alarms by its magnitude will, by being broken up into portions and dealt with separately in each separate district, cease to appear overwhelming or even difficult. Known work being always available for every willing hand, all those who labour among the poor as voluntary helpers or as paid officials will simply have to communicate with the central authority in their own borough, and these again with the heads of departments, and the thing is done. Every unemployed man will thus become known and may be registered; and it will then be impossible for him to migrate from parish to parish ostensibly in search of work, but really in search of the means wherewith to spend his life in more comfortable idleness. If the principles here affirmed were but once thoroughly believed in, and the practical schemes outlined were in full operation, pauperism, street begging, petty crime, discontent, and all forms of misery and wretchedness would be practically extinguished among us. Where is the Hercules who shall cleanse these Augean stables and make our country in this as it has been in so many other respects a model, a leader and an example to the whole civilised world?

